# In-field screening for host plant resistance to *Delia radicum* and *Brevicoryne brassicae* within selected rapeseed cultivars and new interspecific hybrids

**DOI:** 10.1515/biol-2020-0074

**Published:** 2020-09-22

**Authors:** Janetta Niemann, Justyna Szwarc, Jan Bocianowski, Dorota Weigt, Marek Mrówczyński

**Affiliations:** Department of Genetics and Plant Breeding, Poznań University of Life Sciences, Dojazd 11, 60-632, Poznań, Poland; Department of Mathematical and Statistical Methods, Poznań University of Life Sciences, Wojska Polskiego 28, 60-637, Poznań, Poland; Institute of Plant Protection – National Research Institute, Władysława Węgorka 20, 60-318, Poznań, Poland

**Keywords:** *Brassica napus*, rapeseed, pest resistance, hybrids, cabbage root fly, cabbage aphid

## Abstract

Rapeseed (*Brassica napus*) can be attacked by a wide range of pests, for example, cabbage root fly (*Delia radicum*) and cabbage aphid (*Brevicoryne brassicae*). One of the best methods of pest management is breeding for insect resistance in rapeseed. Wild genotypes of *Brassicaceae* and rapeseed cultivars can be used as a source of resistance. In 2017, 2018, and 2019, field trials were performed to assess the level of resistance to *D. radicum* and *B. brassicae* within 53 registered rapeseed cultivars and 31 interspecific hybrid combinations originating from the resources of the Department of Genetics and Plant Breeding of Poznań University of Life Sciences (PULS). The level of resistance varied among genotypes and years. Only one hybrid combination and two *B. napus* cultivars maintained high level of resistance in all tested years, i.e., *B. napus* cv. *Jet Neuf* × *B. carinata* – PI 649096, Galileus, and Markolo. The results of this research indicate that resistance to insects is present in *Brassicaceae* family and can be transferred to rapeseed cultivars. The importance of continuous improvement of rapeseed pest resistance and the search for new sources of resistance is discussed; furthermore, plans for future investigations are presented.

## 1 Introduction

Rapeseed (*Brassica napus* L. ssp. *oleifera* Metzg.) is one of the three most important sources of vegetable oil in the world. The European Union (EU) was the world leader in rapeseed production in 2017 (22 million tons), followed by Canada (21 million tons), China (13 million tons), India (7.9 million tons), Australia (4.3 million tons), and Ukraine (2.1 million tons) [1]. The greatest producers of rapeseed in the EU are France, Germany, Poland, Romania, Great Britain, the Czech Republic, Hungary, Denmark, and Slovakia, respectively [2,3]. Protection from pests is an essential part of breeding programmes – for example, yield losses caused by pests in Poland can range from 15 to 50% [4]. Moreover, a significant increase in the threat from pests is expected, related both to climatic changes and to agrotechnical simplifications [5,6].

Rapeseed plants in Poland are attacked by a wide range of pests. Among them, two economically important insects can be distinguished – cabbage root fly (*Delia radicum* L.) (Diptera: Anthomyiidae) and cabbage aphid (*Brevicoryne brassicae* L.) (Homoptera: Aphididae). The cabbage aphid is one of the most important and commonly occurring insect pests of rapeseed worldwide [7]. *Brevicoryne brassicae* causes significant yield losses in many crops in the family *Brassicaceae*, including mustards and crucifers. Heavy infestation can result in severe plant damage, causing death of seedlings and young transplants. Symptoms in larger plants include curling and yellowing of leaves, stunting of plants, and deformation of developing heads [8,9].

The cabbage root fly is one of the most important pests of many *Brassica* crops in the temperate regions of Europe and North America. After overwintering as pupae and hatching in early spring, females lay eggs in close proximity to the host plant. Depending on the temperature, eggs hatch in about 4 days [8]. The number of generations varies each year from one to four, depending on climatic conditions [10]. Larvae of *D. radicum* can damage plants by feeding on root tissue, resulting in wilting of leaves or the entire plant and eventually reducing the yield and quality of the crop. Moreover, roots attacked by *D. radicum* are more susceptible to secondary root pathogens, such as *Fusarium* spp. [10,11].

To date, three resistance mechanisms have been recognized in the interaction of *Delia*–*Brassica* and *Brevicoryne*–*Brassica*: antixenosis, antibiosis, and tolerance [12]. Antixenosis (non-preference, avoidance) denotes morphological or chemical plant traits that make it unattractive for insects. For example, variation in cabbage leaf colour makes it less attractive to *B. brassicae* [13]. Antibiosis resistance is based on adverse effects of the plant after feeding [14]. Antibiosis does not prevent infestation, but rather causes increased mortality or delayed development of insects. Tolerance means the ability of a plant to reduce inflicted damage. A tolerant host is able to grow and reproduce despite the presence of a high number of insects [12,13]. In contrast to antixenosis and antibiosis, tolerance is independent of the herbivore response but is an adaptive mechanism helping plants to grow normally under biotic stress [15].

For most growers, the use of pesticides is an essential form of protection against harmful organisms [16]. However, there has been an increasing emphasis on the use of environmentally friendly methods of pest control. For example, in 2013, the EU restricted the use of certain neonicotinoids, and in 2018, banned three main neonicotinoids (Commission Implementing Regulation [EU] 2018/783, 2018/784, 2018/785). Moreover, Integrated Pest Management, which focuses on reducing the use of pesticides, has become compulsory for all farmers in the EU since 2014 (Directive 2009/128/EC). Therefore, breeding cultivars with resistance to insect pests fits perfectly into the currently applicable requirements and modern environmentally friendly trends [17,18]. The natural genetic variation among the wild relatives of crop species can provide good sources of novel host plant resistance [19].

Wild and related species of the *Brassicaceae* family are proved to be a valuable source of desirable agronomic traits. For example, *Sinapis alba* has been shown to be tolerant to crucifer flea beetle [20]; *B. juncea*, *B. carinata*, and *B. nigra* can be used to transfer blackleg resistance genes [21]; and *B. rapa*, *B. carinata*, and *S. alba* may act as a source of pod shattering resistance [22]. The assessment of the level of resistance within various *Brassicaceae* wild species or *Brassicaceae* hybrids may help identify genotypes with desired traits, which then can be included into rapeseed breeding programmes.

The aim of this research was to determine the range of pest resistance levels among selected rapeseed cultivars and new *Brassica* hybrid combinations obtained from the Department of Genetics and Plant Breeding of Poznań University of Life Sciences (PULS). This study has been conducted to identify the sources of resistance not only in rapeseed cultivars but also in other brassicaceous species. Consequently, this strategy will allow the assessment of the genetic resistance of interspecific *Brassica* hybrids in comparison with the parental forms in the future.

To the best of our knowledge, this is one of the few studies in which in-field comparison of resistance has been made among rapeseed cultivars and interspecific hybrids towards economically important insect pests.

## 2 Materials and methods

### 2.1 Experimental design

The experiment was conducted for three consecutive years (2017, 2018, and 2019) on the testing fields in PULS experimental station Dłoń (51°41′23″N, 17°04′10″E) located 100 km south from Poznań, Poland. The whole experiment was set up in a completely randomized block design with five replications (on the basis of six plants) in each year (*N* = 90), and each single plot size was 10 m^2^ with a 0.30 row distance and a sowing density of 60 seeds/m^2^. The field experiment in Dłoń was conducted on typical heavy soil of quality class III [23]. Agricultural practices were optimal for local agroecological conditions in Dłoń. Plots were harvested using a plot harvester. In crop seasons 2016/2017, 2017/2018, and 2018/2019, the weather conditions were normal for Poland. The seasonal rainfall in Dłoń was 667 mm in 2017, 372 mm in 2018, and 393 mm in 2019, whereas the mean annual temperatures in 2017, 2018, and 2019 were 9.6, 10.8, and 11.1°C, respectively.

### 2.2 Plant material

Seeds of 53 rapeseed cultivars and 31 hybrid combinations were used as the research material (Table 1). All *Brassica* interspecific hybrids were generated in the Department of Genetics and Plant Breeding of PULS with the application of *in vitro* culture of isolated embryos according to the method described by Niemann et al. [24]. In order to obtain interspecific hybrids with genetic pest resistance, paternal forms harbouring high level of resistance to *B. brassicae* and *D. radicum* were selected according to the literature data.

**Table 1:** List of *Brassicaceae* hybrids and *B. napus* cultivars used as the research material

No. of line	Cross-combination	No. of line	Cross-combination
H1	*B. napus* cv. *Jet Neuf* × *B. rapa* ssp. *pekinensis* 08 007569	H17	*B. napus* cv*. Lisek* × *B. carinata* Dodola
H2	*B. napus* cv. *Jet Neuf* × *B. rapa* ssp. *pekinensis* 08 007574	H18	*B. napus* cv*. Californium* × *B. fruticulosa* – PI649097
H3	*B. napus* cv. *Jet Neuf* × *B. carinata* PI 649091	H19	*B. napus* cv*. Lisek* × *B. fruticulosa* – PI649097
H4	*B. napus* cv*. Górczański* × *B. rapa* ssp. *pekinensis* 08.007574	H20	*B. napus* cv*. Lisek* × *B. fruticulosa* – PI649099
H5	*B. napus* cv*. Górczański* × *B. rapa* ssp. *pekinensis* 08.007569	H21	*B. napus* cv*. Jet Neuf* × *B. carinata* – PI 649094
H6	*B. napus* cv*. Górczański* × *B. rapa* ssp. *Chinensis*	H22	*B. napus* cv. *Jet Neuf* × *B. carinata* – PI 649096
H7	*B. napus* cv*. Lisek* × *S. alba* cv*. Bamberka*	H23	*B. napus* cv*. Californium* × *B. rapa* ssp. *pekinensis* 08 007574-1
H8	*B. napus* cv*. Lisek* × *B. tournefortii*	H24	*B. napus* cv*. Californium* × *B. rapa* ssp. *pekinensis* 08 007574-2
H9	*B. napus* cv. *Lisek* × *B. rapa Pak Choi* 08, 007574	H25	*B. napus* cv*. Californium* × *B. rapa* ssp. *pekinensis* 08 007574-3
H10	*B. napus* cv*. Lisek* × *B. rapa Pak Choi* 08, 007569	H26	*B. napus* cv*. Californium* × *B. rapa* ssp. *pekinensis* 08 007574-4
H11	*B. napus* cv*. Górczański* × *B. rapa Pak Choi* 08, 007574	H27	*B. napus* cv. *Zhongshuang9* × *B. rapa* ssp. *pekinensis* 08 006169
H12	*B. napus* cv*. Jet Neuf* × *B. oleracea var. alboglabra*	H28	*B. napus* MS8 line × *B. rapa* ssp. *pekinensis* 08 006169-1
H13	*B. napus* cv*. Californium* × *B. oleracea var. alboglabra*	H29	*B. napus* MS8 line × *B. rapa* ssp. *pekinensis* 08 006169-2
H14	*B. napus* cv*. Lisek* × *B. oleracea var. alboglabra*	H30	*B. napus* MS8 line × *B. rapa* ssp. *pekinensis* 08 006169-3
H15	*B. napus* cv. *Californium* × *S. alba* cv. *Bamberka*	H31	*B. napus* cv. *Zhongshuang9* × *B. rapa* ssp. *chinensis* 08 007574
H16	*B. napus* cv. *Jet Neuf* × *S. alba* cv. *Bamberka*		

No. of line	Cultivar name	No. of line	Cultivar name
C1	Amir	C28	PX111CL
C2	Inspirati	C29	Anderson
C3	Bufalo	C30	Andromeda
C4	Atora	C31	Arsenal
C5	Dolar	C32	Hybrirock
C6	Fair	C33	Graf
C7	Fantastik	C34	Hary
C8	Jet Neuf	C35	Mickey
C9	Jupiter	C36	150/47
C10	Kana	C37	Prince
C11	Azurio	C38	Sofia
C12	Memoris	C39	Santana
C13	Lindora	C40	Rubin
C14	150/38	C41	Monolit
C15	150/46	C42	Metys
C16	Walegro	C43	Chrobry
C17	Marita	C44	150/42
C18	150/40	C45	Kabriolet
C19	150/44	C46	Falcon
C20	Razmus	C47	Diger
C21	Walery	C48	Corina
C22	Aruze	C49	Kontakt
C23	Bazyl	C50	Ceres
C24	Bellinda	C51	Galileus
C25	Californium	C52	Markolo
C26	Darmor	C53	Hewelius
C27	PR48W26		

All interspecific cross-derived lines were sister-pollinated (five plants were enclosed in one paper bag during flowering) for four generations in order to stabilize the fertility [25]. Morphotypes of plants of the F_5_–F_7_ generations were compared with the parental lines, as described by Wojciechowski [26]. Analysis of selected morphological traits was performed in order to determine whether the obtained plants resembled the *B. napus* type or the paternal type. The examination was based on (a) leaf colour, (b) presence of trichomes on the lower side of the leaf blade, (c) position of the buds relative to the open flowers, (d) growth habit, (e) type of inflorescence, and (f) flower characteristics (sterile or fertile).

### 2.3 Assessment of pest resistance

The assessment of pest resistance was carried out for two insects (*Delia radicum* and *Brevicoryne brassicae*) and consisted of plant damage evaluation. General damage by insects was assessed at the end of the season, in late October 2017, 2018, or in early November 2019 in Dłoń. All assessments, i.e., direct damage on roots for *D. radicum* and on leaves for *B. brassicae*, were performed according to the EPPO standards [27] on randomly chosen individuals. For every genotype, six plants were assessed. The severity of insect damage on plants was evaluated at physiological maturity on a 1 to 9 scoring scale, used commonly by the Research Centre for Cultivar Testing in Poland, which corresponds with the International Union for the Protection of New Varieties of Plants [28] system of assessment. According to this scale, score 9 means no visible damage on plants (highly resistant), and score 1 means a completely damaged plant (fully susceptible) (Table 2). No pesticides were used on the plots. The average values from six plants were calculated for each replication. In this way, we obtained quantitative trait data with normal distributions.

**Table 2:** Insect pest damage rating scale. Visual symptoms observed on roots (*Delia radicum*) or on leaves (*Brevicoryne brassicae*)

Scale	Visual symptoms	Plant response
1	Lesions profuse on 100% of the roots and leaf surface	Susceptible
2	Lesions present on up to 90% of the roots and leaf surface	Susceptible to moderately susceptible
3	Lesions present on up to 70–75% of the roots and leaf surface	Moderately susceptible
4	Lesions visible on up to 50% of the roots and leaf surface	Moderately susceptible to moderately resistant
5	Lesions visible on up to 25% of the roots and leaf surface, little damage	Moderately resistant
6	Lesions visible on less than 15–20% of the roots and leaf surface	Moderately resistant to resistant
7	Lesions visible on less than 10% of the roots and leaf surface	Resistant
8	Lesions visible on less than 5% of the roots and leaf surface	Resistant to highly resistant
9	No insect damage visible on any analysed part of the plant	Highly resistant

### 2.4 Statistical analysis

The normality of the distributions of the studied traits (resistance to *B. brassicae* and resistance to *D. radicum*) was tested using the Shapiro–Wilk normality test [29]. Two-way analyses of variance (ANOVA) with blocks were carried out to determine the effects of year, genotype (cultivars and hybrids, independently), and year × genotype interaction on the variability of resistance to *B. brassicae* and resistance to *D. radicum*. The mean values and standard deviations of the observed traits were calculated for each genotype in all years of study. Fisher’s least significant differences (LSDs) were estimated for individual traits, and on this basis, homogeneous groups were determined. Differences between cultivars and hybrids were tested on the basis of a *t*-test, independently for resistance to *B. brassicae* and resistance to *D. radicum*. We used the critical significance level equal to 0.05, resulting from a Bonferroni correction. All the analyses were conducted using the GenStat v. 18 statistical software package (VSN International, Hemel Hempstead, UK).

## 3 Results

### 3.1 Morphology of hybrid plants

The individual interspecific and intergeneric hybrid combinations of F_5_–F_7_ generations had reasonably uniform morphological characteristics. Moreover, plants of all tested lines were very consistent in growth habit. Hybrid plants obtained from crosses between *B. napus* × *B. rapa* genotypes were similar to rapeseed. However, in a small number of cases, some morphological features were similar to those of turnip rape, e.g., lighter leaf colour, trichomes on the lower side of the leaf blade, and turnip rape-like inflorescence. No significant new characteristics, absent in either parent, were reported in the hybrids. All other hybrid plants resembled more paternal morphotypes. Consequently, plants obtained from crosses between *B. napus* × *B. carinata*, *B. juncea*, and *S. alba* genotypes had young leaf surfaces with high trichome density.

### 3.2 Assessment of pest resistance

The results of the ANOVA indicated that the effects of cultivar, hybrid, and year were significant for both tested traits (resistance to *B. brassicae* and *D. radicum*). The year × genotype interactions were highly significant for both observed traits for cultivars and hybrids (Table 3).

**Table 3:** Mean squares (m.s.) from two-way analysis of variance for *Brevicoryne brassicae* and *Delia radicum* (hybrid and cultivar resistance) (*N* = 90)

Source of variation	*Brevicoryne brassicae*			*Delia radicum*		
	d.f.	m.s.	*p*-Value	d.f.	m.s.	*p*-Value
**Hybrids**						
Block	4	0.73		4	1.27	
Hybrid	30	2.7592	<0.001	30	20.438	<0.001
Year	2	241.1076	<0.001	2	18.884	0.022
Hybrid × year	57	3.3161	<0.001	57	12.488	<0.001
Residual	425	0.5328		427	4.875	
**Cultivars**						
Block	4	0.91		4	1.32	
Cultivar	52	5.9015	<0.001	52	30.982	<0.001
Year	2	1074.9311	<0.001	2	290.038	<0.001
Cultivar × year	104	7.7494	<0.001	104	23.986	<0.001
Residual	897	0.4831		632	4.339	

d.f.  – the number of degrees of freedom.

The mean values of resistance to insect pests for the analysed hybrids and cultivars in the years studied successively, i.e., 2017, 2018, and 2019, are presented in Table 4. In general, the resistance to both pests varied among years. The highest mean level of resistance to *B. brassicae* was observed for cultivars in 2017 (8.991), whereas the lowest in 2018 was also for cultivars (5.513). For *D. radicum*, the highest mean resistance was noticed in 2019 for hybrids (7.153). In contrast, the lowest mean resistance was observed for cultivars in 2017 (4.136).

**Table 4:** Mean resistance to *Brevicoryne brassicae* and resistance to *Delia radicum* (and standard deviations) of all investigated *Brassica napus* cultivars and hybrid lines over three years

	2017		2018		2019	
	Hybrids	Cultivars	Hybrids	Cultivars	Hybrids	Cultivars
**Resistance to *Brevicoryne brassicae***						
Number of observations	309	530	93	265	117	265
Mean	8.803	8.991	6.28	5.513	7.692	7.57
Standard deviation	0.5494	0.0968	1.913	2.326	0.6881	0.6599
*t*-Statistic	−5.96		3.13		1.65	
*p*-Value	<0.001		0.002		0.1	
**Resistance to *Delia radicum***						
Number of observations	310	265	93	265	118	265
Mean	6.697	4.136	6.581	5.804	7.153	5.362
Standard deviation	2.617	2.568	3.076	3.034	1.556	2.537
*t*-Statistic	11.8		2.12		8.46	
*p*-Value	<0.001		0.035		<0.001	

The obtained data showed that the level of pest resistance varied between cultivars and hybrids. Compared to the analysed cultivars, the mean resistance of hybrid plants was higher in all tested years for *D. radicum*. For *B. brassicae*, the mean resistance of hybrids was higher only in 2018. The difference in resistance to *B. brassicae* among cultivars and hybrids in 2019 was not statistically significant (Table 5).

**Table 5:** Mean values and standard deviations (s.d.) for hybrid resistance to *Brevicoryne brassicae* and resistance to *Delia radicum* (*N* = 90)

Hybrid	Resistance to *Brevicoryne brassicae* (9° scale)						Resistance to *Delia radicum* (9° scale)					
	2017		2018		2019		2017		2018		2019	
	Mean	s.d.	Mean	s.d.	Mean	s.d.	Mean	s.d.	Mean	s.d.	Mean	s.d.
H1	8.8abc	0.42	6.333bcdef	0.58	6.667c	2.31	6.9bcde	2.56	8.333ab	0.58	3.667i	3.79
H2	9a	0.00	6.333bcdef	1.15	7.8ab	0.45	6.5bcdef	3.38	7.333abc	2.89	8abc	1.23
H3	8.8abc	0.42	7abcd	0.00	7.333bc	0.58	7.6abc	1.58	6abc	4.36	7.333abcd	1.16
H4	8.889ab	0.33	3i	1.73	6.667c	1.53	7.3abc	2.41	6abc	3.46	7.667abc	0.58
H5	9a	0.00	5.333cdefgh	1.53	7.4abc	1.34	7.2abcd	2.78	7abc	1.73	6.6cdef	2.07
H6	9a	0.00	3.333hi	2.31	7.8ab	0.45	7.4abc	2.80	3.667cd	3.79	7.4abcd	0.55
H7	8.8abc	0.42	3.667ghi	2.89	7.333bc	0.58	5.8cdef	3.08	4bcd	4.36	7abcde	1.00
H8	8.5bcd	0.71	6bcdef	1.73	8ab	0.00	6.1cdef	2.64	6abc	2.65	8.4a	0.55
H9	8.7abcd	0.48	5defghi	1.00	7.667ab	0.58	6.6bcdef	2.27	4bcd	1.73	6defg	2.00
H10	8.5bcd	0.85	7.333abc	0.58	7.8ab	0.45	6.5bcdef	2.55	9a	0.00	6.8bcdef	1.64
H11	8.7abcd	0.48	7abcd	0.00	8ab	0.00	3.5gh	2.92	9a	0.00	7.8abc	0.84
H12	9a	0.00	4.333fghi	2.52	8ab	0.00	6.6bcdef	3.37	5.667abc	4.04	8.333ab	0.58
H13	8.9ab	0.32	7.333abc	0.58	8ab	0.00	4.9efg	1.85	5.333abcd	3.79	8.2ab	0.45
H14	8.8abc	0.63	7.667ab	0.58	7.25bc	0.96	6.9bcde	2.85	6.667abc	3.22	7.5abcd	1.00
H15	8.6abcd	0.70	4.333fghi	3.06	7.8ab	0.45	7.3abc	1.42	1d	0.00	7.6abc	0.55
H16	9a	0.00	6.667bcde	0.58	7.333bc	0.58	6.8bcde	3.55	5.667abc	4.04	5ghi	1.73
H17	8.9ab	0.32	6.333bcdef	2.08	8.2a	0.45	7.1abcd	2.18	6.667abc	3.22	7.8abc	0.84
H18	8.7abcd	0.95	7abcd	0.00	8ab	0.00	6.3cdef	1.57	5.667abc	4.04	7.4abcd	0.89
H19	8.4cd	0.70	5.333cdefgh	0.58	7.6ab	0.55	2.3h	1.57	6abc	4.36	7.2abcde	1.30
H20	8.9ab	0.32	5.667bcdefg	1.15	7.6ab	0.55	8.4ab	0.84	5.667abc	4.04	7.8abc	0.84
H21	9a	0.00	6bcdef	1.00	8ab	0.00	7.4abc	2.17	6abc	4.36	6.8bcdef	1.30
H22	9a	0.00	7.333abc	1.15	8ab	0.00	7.8abc	2.04	8.333ab	0.58	7.8abc	0.45
H23	7.9e	1.20	4.667efghi	2.08	7.5abc	0.58	4.6fg	1.71	5.667abc	4.04	7.5abcd	0.58
H24	8.3de	1.16	7.667ab	0.58	8ab	0.00	5.2defg	1.32	8.333ab	0.58	5.333fgh	1.53
H25	8.8abc	0.63	6.333bcdef	1.15	8ab	0.00	6.3cdef	2.16	3.667cd	2.89	7.8abc	0.45
H26	9a	0.00	7.667ab	0.58	7.333bc	0.58	7.7abc	2.58	9a	0.00	5.667efg	1.53
H27	9a	0.00	6.667bcde	0.58	7.667ab	0.58	7.5abc	2.59	8.333ab	1.16	4hi	2.65
H28	9a	0.00	9a	0.00	–	–	9a	0.00	9a	0.00	–	–
H29	9a	0.00	9a	0.00	–	–	9a	0.00	9a	0.00	–	–
H30	9a	0.00	9a	0.00	–	–	9a	0.00	9a	0.00	–	–
H31	9a	0.00	6.333bcdef	1.15	7.4abc	0.89	6.1cdef	1.91	9a	0.00	7.4abcd	0.89
LSD_0.05_	0.45		2.233		0.841		2.01		4.644		1.592	

Values with different letters in columns are significantly different.

More detailed results are presented in Tables 5 and 6. The conducted analyses showed significant differences between the tested plants. Moreover, the collected data allowed us to distinguish a group of genotypes with the highest resistance to pests (belonging to group *a*) in tested years for both hybrids and cultivars. Within those plants, we found individuals that belonged to statistically the best group for all three successive years (Table 7). Four hybrids (e.g., *B. napus* cv. *Górczański* × *B. rapa* Pak Choi 08, 007574) and 27 cultivars (e.g., Inspirati) maintained the high level of resistance to *B. brassicae* during the tested years. However, only five hybrids (e.g., *B. napus* cv. *Jet Neuf* × *B. carinata* PI 649091) and two rapeseed cultivars (Galileus and Markolo) maintained the high level of resistance to *D. radicum*. Among the tested plant genotypes, only one hybrid and two cultivars remained resistant for both pests in three years, i.e., *B. napus* cv. *Jet Neuf* × *B. carinata* – PI 649096, Galileus, and Markolo.

**Table 6:** Mean values and standard deviations (s.d.) for cultivar resistance to *Brevicoryne brassicae* and resistance to *Delia radicum* (*N* = 90)

Cultivar	Resistance to *Brevicoryne brassicae* (9° scale)						Resistance to *Delia radicum* (9° scale)					
	2017		2018		2019		2017		2018		2019	
	Mean	s.d.	Mean	s.d.	Mean	s.d.	Mean	s.d.	Mean	s.d.	Mean	s.d.
C1	9a	0.00	4.6ghi	2.19	8a	0.00	6.2bcde	1.64	6.6abcdefg	1.67	7abcdef	1.00
C2	9a	0.00	6.8abcd	0.84	7.4abcd	0.89	3.4ijklmno	2.88	5.8abcdefghijk	2.95	7.4abcd	0.89
C3	9a	0.00	6.2abcdefg	1.10	8a	0.00	7b	1.23	3.4hijklmn	3.36	7.6abc	0.55
C4	9a	0.00	6.8abcd	1.10	7.8ab	0.45	4ghijklmn	2.12	5.8abcdefghijk	2.95	7.2abcde	0.84
C5	9a	0.00	5.4cdefg	1.14	7.4abcd	0.55	5.2cdefgh	2.49	4.6defghijklm	3.29	1.2n	0.45
C6	8.9a	0.32	7.2ab	0.45	7.6abc	0.55	5defghi	2.45	5.2cdefghijkl	3.83	3jklmn	1.87
C7	9a	0.00	6.2abcdefg	0.45	7.6abc	0.55	5.6bcdefg	0.55	6abcdefghij	2.92	6.4abcdefg	2.51
C8	9a	0.00	7.6a	0.55	7.6abc	0.55	3.4ijklmno	1.95	8.2abc	0.45	5.4bcdefghij	2.70
C9	9a	0.00	6.6abcde	0.55	7.6abc	0.55	4.8defghij	1.30	8abc	0.71	3.6hijklmn	3.13
C10	9a	0.00	7.6a	0.55	7cde	0.00	2.2opqr	0.84	7abcdef	3.39	1.6n	0.55
C11	9a	0.00	5.2defgh	0.45	6.6de	1.52	2opqr	2.24	3jklmn	3.08	3.2ijklmn	2.17
C12	9a	0.00	7abc	0.71	7.6abc	0.55	1.2qr	0.45	7abcdef	2.24	7.4abcd	0.55
C13	9a	0.00	7.2ab	0.84	7.8ab	0.45	3klmnop	2.35	7.2abcde	3.49	5.8abcdefgh	2.39
C14	8.9a	0.32	5.2defgh	0.45	7.8ab	0.45	4.6efghijk	2.30	3jklmn	2.35	6.2abcdefg	1.64
C15	9a	0.00	5.2defgh	1.30	7.4abcd	0.89	1.4pqr	0.55	5.8abcdefghijk	3.11	6.2abcdefg	1.64
C16	8.9a	0.32	7.4ab	0.89	7.6abc	0.55	3.2jklmno	2.17	8.4ab	0.55	5defghijkl	3.00
C17	8.9a	0.32	7abc	1.00	7cde	0.00	2.4nopqr	1.34	5.2cdefghijkl	3.03	3.6hijklmn	3.21
C18	9a	0.00	7abc	1.22	7.8ab	0.45	1r	0.00	8.6ab	0.55	5.6bcdefghi	2.79
C19	9a	0.00	4.8fghi	1.10	7.6abc	0.55	4.6efghijk	3.51	6.2abcdefghi	3.03	2.2mn	2.17
C20	9a	0.00	2.4jkl	1.52	6.8de	1.10	4.4fghijkl	2.88	5.8abcdefghijk	2.78	2.6lmn	1.95
C21	9a	0.00	2.4jkl	1.52	7.6abc	0.55	4.2fghijklm	1.92	2.8klmn	2.05	5.4bcdefghij	2.88
C22	8.9a	0.32	2jkl	1.22	8a	0.00	3.6hijklmno	2.30	7.6abcd	0.55	6.4abcdefg	0.89
C23	9a	0.00	3.2ijk	2.68	7.8ab	0.45	4.6efghijk	2.07	7.4abcde	1.52	6.6abcdefg	2.61
C24	9a	0.00	2.8jk	1.10	7.4abcd	0.55	1.4pqr	0.55	7.8abc	0.45	2.8klmn	2.39
C25	9a	0.00	2.8jk	2.05	8a	0.00	5.4bcdefg	0.55	4fghijklmn	3.00	3.6hijklmn	2.30
C26	9a	0.00	1l	0.00	6.6e	1.52	2.8lmnopq	2.17	1.2n	0.45	4.8efghijkl	1.92
C27	9a	0.00	1.8kl	1.30	6.6e	1.52	3.2jklmno	1.92	2.2lmn	1.79	2.2mn	1.10
C28	9a	0.00	1l	0.00	7.2bcde	0.45	4.4fghijkl	1.95	2mn	1.00	6.2abcdefg	1.64
C29	9a	0.00	2.8jk	2.05	7.6abc	0.55	2.4nopqr	1.52	8abc	0.00	2.6lmn	1.34
C30	9a	0.00	3.6hij	3.58	7.6abc	0.55	4ghijklmn	2.74	4fghijklmn	3.74	7.2abcde	0.84
C31	9a	0.00	6.4abcdef	0.89	7.6abc	0.55	3klmnop	2.74	6.8abcdefg	2.68	1.2n	0.45
C32	9a	0.00	2.8jk	1.30	7.2bcde	0.45	3.6hijklmno	1.67	3.8ghijklmn	3.42	6.4abcdefg	1.14
C33	9a	0.00	6.6abcde	1.67	7.8ab	0.45	5.6bcdefg	1.14	2.4lmn	2.61	7abcdef	1.23
C34	9a	0.00	7.8a	0.45	7.6abc	0.55	3.6hijklmno	2.07	8.6ab	0.55	7.6abc	0.89
C35	9a	0.00	7.4ab	0.55	7.4abcd	0.89	2.6mnopqr	2.07	8.4ab	0.55	5.2cdefghijk	0.84
C36	9a	0.00	7.2ab	0.84	7.4abcd	0.55	4.6efghijk	3.29	4.6defghijklm	3.51	5.6bcdefghi	2.07
C37	9a	0.00	7.6a	0.55	7.2bcde	0.45	1.4pqr	0.55	8.6ab	0.55	4.4ghijklm	1.95
C38	9a	0.00	5.8bcdefg	0.45	7.6abc	0.55	7b	1.00	3.2ijklmn	2.95	5.6bcdefghi	3.29
C39	9a	0.00	6.8abcd	0.45	7.4abcd	0.55	3.2jklmno	1.92	8abc	0.71	7.4abcd	0.55
C40	9a	0.00	3.6hij	2.07	7.4abcd	0.89	1.4pqr	0.55	5.2cdefghijkl	2.39	6abcdefgh	1.00
C41	9a	0.00	7abc	0.00	7.8ab	0.45	2.2opqr	1.30	7.8abc	1.64	8.2a	0.45
C42	9a	0.00	5.2defgh	0.45	8a	0.00	4.4fghijkl	1.67	1n	0.00	4.8efghijkl	2.59
C43	9a	0.00	7.4ab	0.55	7.6abc	0.55	4ghijklmn	2.55	7.8abc	0.45	6abcdefgh	2.24
C44	9a	0.00	6.4abcdef	1.14	8a	0.00	2.2opqr	1.30	3.8ghijklmn	3.03	6.6abcdefg	2.61
C45	9a	0.00	2.6jkl	2.51	7.8ab	0.45	5.4bcdefg	2.41	5.6bcdefghijk	3.44	6.6abcdefg	1.67
C46	9a	0.00	5efgh	2.00	8a	0.00	3.6hijklmno	2.30	7abcdef	2.83	6.4abcdefg	2.51
C47	9a	0.00	7.2ab	1.10	8a	0.00	4.8defghij	2.59	6.2abcdefghi	2.68	4.8efghijkl	2.28
C48	9a	0.00	6.2abcdefg	1.79	7.8ab	0.45	6.4bcd	0.89	4.4efghijklm	3.29	6abcdefgh	1.41
C49	9a	0.00	7.6a	0.55	8a	0.00	5.8bcdef	1.10	8.8a	0.45	5.2cdefghijk	2.68
C50	9a	0.00	6.4abcdef	0.89	7.6abc	0.55	6.8bc	1.30	6.4abcdefgh	2.07	7.8ab	0.45
C51	9a	0.00	7.2ab	0.45	8a	0.00	9a	0.00	7.8abc	0.84	7abcdef	1.87
C52	9a	0.00	7.4ab	1.52	7.6abc	0.55	9a	0.00	6.6abcdefg	2.61	7.8ab	0.84
C53	9a	0.00	7.8a	0.84	8a	0.00	9a	0.00	7abcdef	3.39	4.6fghijklm	2.51
LSD_0.05_	0.085		1.61		0.76		1.65		3		2.4	

Values with different letters in columns are significantly different.

**Table 7:** List of genotypes with high resistance to pests in three successive years

*Brevicoryne brassicae*	
Hybrids	H11*, H13, H18, **H22**
Cultivars	C2, C3, C4, C6, C7, C8, C9, C12, C13, C16, C18, C31, C33, C34, C35, C36, C39, C41, C43, C44, C47, C48, C49, C50, **C51, C52**, C53
*Delia radicum*	
Hybrids	H3, H4, H17, H20, **H22**
Cultivars	**C51, C52**

Genotypes resistant to both pests are highlighted in bold font.

* ^*^Numbers according to Table 1.

## 4 Discussion

As stated before, in recent years, the use of insecticides became partly limited – some chemicals have been withdrawn due to their harmful effects on the environment. It causes many problems for farmers, as the range of effective insecticides is getting narrowed. Moreover, the use of chemicals may not always be successful as insects can develop resistance. For both insects, i.e., *D. radicum* and *B. brassicae*, cases of resistance to certain pesticides have been reported [30,31,32]. Considering this, host plant resistance might be the future of pest management, as it is one of the most economically feasible and ecologically sustainable options [33]. Several strategies to obtain insect-resistant rapeseed have been already presented [34]. This study has successfully followed two of them: finding the source of resistance within *Brassicaceae* species and selecting the insect-resistant rapeseed cultivars among cultivars that have been already registered.

Previous studies showed that wild species of *Brassicaceae* can be a useful source of resistance to *B. brassicae* and *D. radicum*. For example, *B. fruticulosa* and *B. spinescens* have a very high level of resistance to both pests and may be used as research material to find respective Quantitative Trait Loci (QTLs) or as part of a breeding programme [35,36]. Moreover, Dosdall et al. [37] screened many genotypes within *Brassicaceae* and successfully produced *S. alba* × *B. napus* hybrids that inherited resistance to *Delia* spp. from *S. alba*.

However, according to the literature data, much uncertainty still exists about insect feeding preferences and sources of plant resistance to pests [38]. Despite this, there is a considerable amount of literature comparing the life history traits of adults and larvae of pollen beetles among species of *Brassicaceae* [39,40,41]. For example, *S. alba* may act as a donor of resistance, which can be successfully introgressed into rapeseed. Moreover, *S. alba* genotypes show resistance to a few other pests of rapeseed: root flies *Delia* spp. [37,42], flea beetle *P. cruciferae* [43,44], and bertha armyworm *Mamestra configurata* [45]. However, based on the in-field screening performed in this study, it is not possible to confirm that the obtained *B. napus* × *S. alba* hybrid combinations were able to maintain higher level of resistance to *D. radicum* or *B. brassicae* during the three consecutive years of study. Furthermore, review of the literature supports resistance to pollen beetles also in *Eruca sativa* [40] and in *C. abyssinica* [46].

Breeding programmes depending on resistant materials are presently also being applied against *Ceutorhynchus obstrictus* (Marsham) (Coleoptera: Curculionidae). Previous experience in other countries has shown that among the tested *Brassicaceae* species, the white mustard *S. alba* was much less susceptible than rapeseed to *C. obstrictus* damage [47].

These studies confirm our assumption that some of the interspecific or intergeneric hybrids can be successfully used as part of future breeding strategies.

Generally, rapeseed cultivars are not considered a very promising source of resistance to pests, as screenings for resistance within existing varieties rarely bring expected results [38,48,49]. Despite this, we managed to find genotypes within *B. napus* (Galileus and Marcolo), which are moderately or highly resistant to both *B. brassicae* and *D. radicum*. Our observations have shown that in the future more assessments should be performed to verify a greater number of cultivars.

Our research has proven the existence of insect-resistant genotypes among rapeseed cultivars and *Brassicaceae* hybrids. A few genotypes were able to maintain the high level of resistance in the three consecutive years of field experiments, which seems to be very useful in future insect resistance breeding. Observed differences in the infestation level allow us to conclude that the plant response might be conditioned by genotype, which may give a chance to identify resistance genes. Future work should focus on laboratory studies, to determine the genetic basis of resistance, as it may depend on three systems: antixenosis, antibiosis, or tolerance [35]. Moreover, research conducted by Hao et al. [50] showed that aphids have preferential behaviour regarding the host plant. Upper epidermis thickness and trichome length had significant impact on aphids’ preference on initial probing, which leads to a conclusion that physical properties of rapeseed leaves may be important for *B. brassicae* host choice.

The level of plant damage varied over the years of observation. Therefore, it can be concluded that the results of the field trials might have been partly dependent on the weather or other abiotic and biotic stresses [34]. Population dynamics of insects may be affected by parameters such as temperature, humidity, and total rainfall [51,52]. Many factors affect the plant response to insects, which makes it harder to find individuals with true genetically induced resistance to insects.

Currently, insect resistance research is focused on quantitative resistance, as it might provide a more durable effect than pyramiding single resistance genes [34]. Variability of insect-derived damage observed in our study proves the complexity of plant response to pests. This might indicate that the resistance of tested genotypes relies on multiple genes located in QTLs. This type of resistance is usually harder to track, because of its complexity and dependence on environmental factors [53]. This makes quantitative traits difficult to include in breeding programmes. However, a study by Ekuere et al. [54] proves that it is possible to track QTLs conferring resistance to *Delia* spp. by using linkage analysis. Successful introduction of multigenic resistance to insects in *Brassica* crops would be a great strategy in pest management.

In conclusion, we found several sources of resistance to *D. radicum* and *B. brassicae* among the rapeseed cultivars, i.e., Galileus and Marcolo, and interspecific *Brassicaceae* hybrids, i.e., *B. napus* cv. *Jet Neuf* × *B. carinata* – PI 649096. Some of the genotypes showed high level of resistance over the three successive years of field trials. These genotypes are especially valuable and should be diligently analysed.
